# The Changing Landscape of Global Biopharmaceutical Leadership, 2004–2024

**DOI:** 10.1007/s43441-026-01031-w

**Published:** 2026-08-07

**Authors:** Yevgeniy Feyman, John M. O’Brien, Jonathan D. Campbell

**Affiliations:** National Pharmaceutical Council, 1717 Pennsylvania Ave NW #800, Washington, DC 20006 USA

**Keywords:** Pharmaceutical policy, Most favored nation, Innovation, Drug pricing, Leadership

## Abstract

For several decades, the United States (U.S.) has been the leader in biopharmaceutical advancements. Recently, the U.S. has increased government interventions to limit prices for patent-protected medicines. While it is unclear whether ongoing efforts to implement international reference pricing in the U.S. could have similar effects, descriptive measures of global biopharmaceutical market dynamics can help benchmark future assessments. In this analysis, we analyzed trends in the geographic distribution of select measures of biopharmaceutical leadership from 2004 through 2024. Using the PharmaProjects database from Citeline, we found that 43.1% of first launches of small-molecule and biologic drugs occurred in the U.S., 15.3% occurred in Europe, and 9.9% occurred in China. Over this time, the share of drugs launched first in the U.S. went from 45.9 to 37.8%, the share of drugs launched first in Europe fell from 26.7 to 8.5%, while this share in China grew from 3.3 to 28.7%. The share of first-launched therapeutics developed by U.S.-headquartered companies and by companies based in Europe declined while the share developed by companies based in China increased. Though additional research is needed on the measurement of biopharmaceutical leadership, and impacts of government intervention, this descriptive study suggests that over several decades, there has been a strong decline in European measures of leadership. At the same time, China is becoming increasingly important in the global biopharmaceutical space. This analysis supports the view that the U.S remains a leader, and offers a benchmark and context for future evaluations that consider potential unintended consequences of ongoing proposals to increase government intervention in the prices of patent-protected medicines.

## Introduction

Prior to the 1990s, European countries were the primary source of global pharmaceutical innovation and predominantly where pharmaceutical products were first launched. From 1982 to 1992, more than half of all new chemical entities (NCEs) were launched first in Europe, with fewer than 10% of first launches occurring in the U.S. Over the same period, nearly 50% of all NCEs were developed by countries headquartered in the EU, while only about one-quarter came from companies headquartered in the U.S. [1]. This trend was largely consistent in the decades prior as well [2].

These trends changed in the 1990s. Europe accounted for 34.6% of first launches from 1993 to 2003, with 41.9% of NCEs developed by European-headquartered firms. The U.S. share of first launches grew to 35.7%, nearly proportional to the share of NCEs developed by U.S.-headquartered firms (34.8%) [1].

Evidence from independent researchers, the European Commission, and the Government Accountability Office (GAO) has found that this decline is in part attributed to the use of policies (such as price controls and reference pricing) that regulate pharmaceutical pricing and profits [3–7].

Two analyses examined the distribution of biopharmaceutical innovation globally for more recent years. First, analyses by RAND focused on first-launches [8], but was limited to drugs launched between 2018 and 2022 and focused solely on the U.S. and Organization for Economic Cooperation and Development (OECD) countries. Second, an analysis by Kinch et al. [9] used ClinicalTrials.gov data to assess where developers of active pharmaceutical ingredients (APIs) have been located over time. While this included recent years of data, it focused solely on developer headquarters location rather than where biopharmaceuticals were being first launched. In addition, ClinicalTrials.gov is substantially less complete as compared to data collected by Citeline [10]. Related work has investigated time-to-launch differences in new molecular entities between the U.S. and China, finding that time-to-launch in recent years has been similar, with time-to-launch in China falling over time [11, 12].

Existing evidence indicates has demonstrated unintended consequences from the Inflation Reduction Act (IRA) on patient access to new therapeutics [13, 14]. It is unclear if ongoing efforts to increase government intervention in the U.S. biopharmaceutical market (for instance, through mandatory international reference pricing) will have deleterious effects. However, future evaluations of such efforts will require an up-to-date benchmark from which to make assessments; updated evidence on the distribution of market launches and development location is critical to inform policymakers, regulators, and to support future research.

Thus, in this analysis, we examine small-molecule and biologic drug first launches from 2004 through 2024. We characterize the first-launched markets, the headquarters location of the developing firm, and examine trends by disease category.

## Materials and Methods

### Drug Inclusion

We used the Citeline PharmaProjects [15] database to identify all drugs with a first-launch date between January 1, 2004 and December 31, 2024. The study sample was restricted to biologic and small-molecule drugs and allowed for reformulation of drugs as well (e.g., tablet vs. liquid or extended-release versions). We excluded certain drugs such as those that are strictly diagnostic or imaging agents, those that are only categorized as natural products (e.g., Traditional Chinese Medicine), or those for which the drug type was not identified. The sample excluded small-molecule generics considered therapeutically equivalent to existing drugs but included biosimilar products.

These inclusion and exclusion criteria represent new molecules or targets as well as follow-on innovations (such as combination therapies, which are captured as “reformulations” in our data). Follow-on innovations expand patient access. Therefore, investment in these developments reflects market characteristics that help to support the biopharmaceutical sector, and hence, may reflect leadership in biopharmaceuticals.

In a sensitivity analysis, we restricted our sample to drugs categorized as new active substances (NASs) by Citeline. This may help address potential issues with identifying novel drugs developed by firms headquartered in China. However, our primary focus is broad and is intended to capture all improvements in medicines.

### Drug Categorization

Where Citeline’s categorization of biologic versus small molecule included both categories, we used DrugBank.com and identified the modality. For remaining products where molecular classification is ambiguous—particularly oligonucleotides, peptides, and other complex molecules that do not fit neatly into traditional small or large molecule categories—we deferred to the FDA regulatory approval pathway, classifying products approved under a New Drug Application (NDA) as small molecule and those approved under a Biologics License Application (BLA) as biologics.

### Country Identification

While Citeline data identified the location of the HQ of the developing firm for a drug, the country of first launch was typically located in a free text field. To identify the country of first launch, we parsed this field for observations where the event type indicated a first launch. Additionally, we mapped the originator firm and their headquarters to the drug, excluding licensees. We further consolidated European countries into a single region (“Europe”). When multiple European countries were listed as launch countries, we assigned the region as “Europe.” When multiple other non-European countries were listed, we assigned the region as “Multiple.” Lastly, when European and non-European countries were listed, we also assigned the region as “Multiple.” When the launch country could not be identified, we assigned the region as “Unknown.” These represented a small share of the sample—2.3% of first launches were “Unknown,” since they were missing the launch country, and another 2.3% were listed as “multiple” countries—suggesting they were unlikely to substantially affect our primary findings.

### Disease Category Identification

When identifying disease categories, we relied on the first listed category. Although some drugs may have multiple categories due to approvals across multiple indications, we assigned each drug to a single category. Because there were many disease categories, we only summarized disease categories with more than 300 drugs launched over the two decades of data. These categories included: anti-infectives, neurological drugs, and oncology drugs. All others were grouped into an “other” category.

### Alternative Measures of Leadership

We note that there are numerous dimensions and measures of biopharmaceutical leadership. For instance, patent activity and scientific output are often considered indicators of innovation. Other measures might focus on regulatory processes and investigate all approval activity, or even approvals within a subset of disease categories or drug types. Our chosen measures, which focus on where products are first launched and the headquarters location of the firm, are a subset of leadership measures linked to patient access and investment in a country. Though a subset of biopharmaceutical leadership, the measures highlight an important dynamic—that biopharmaceutical leadership should be viewed in the context of whether patients are able to access the resulting innovations.

## Results

### First Launches Overall

**Fig. 1:**
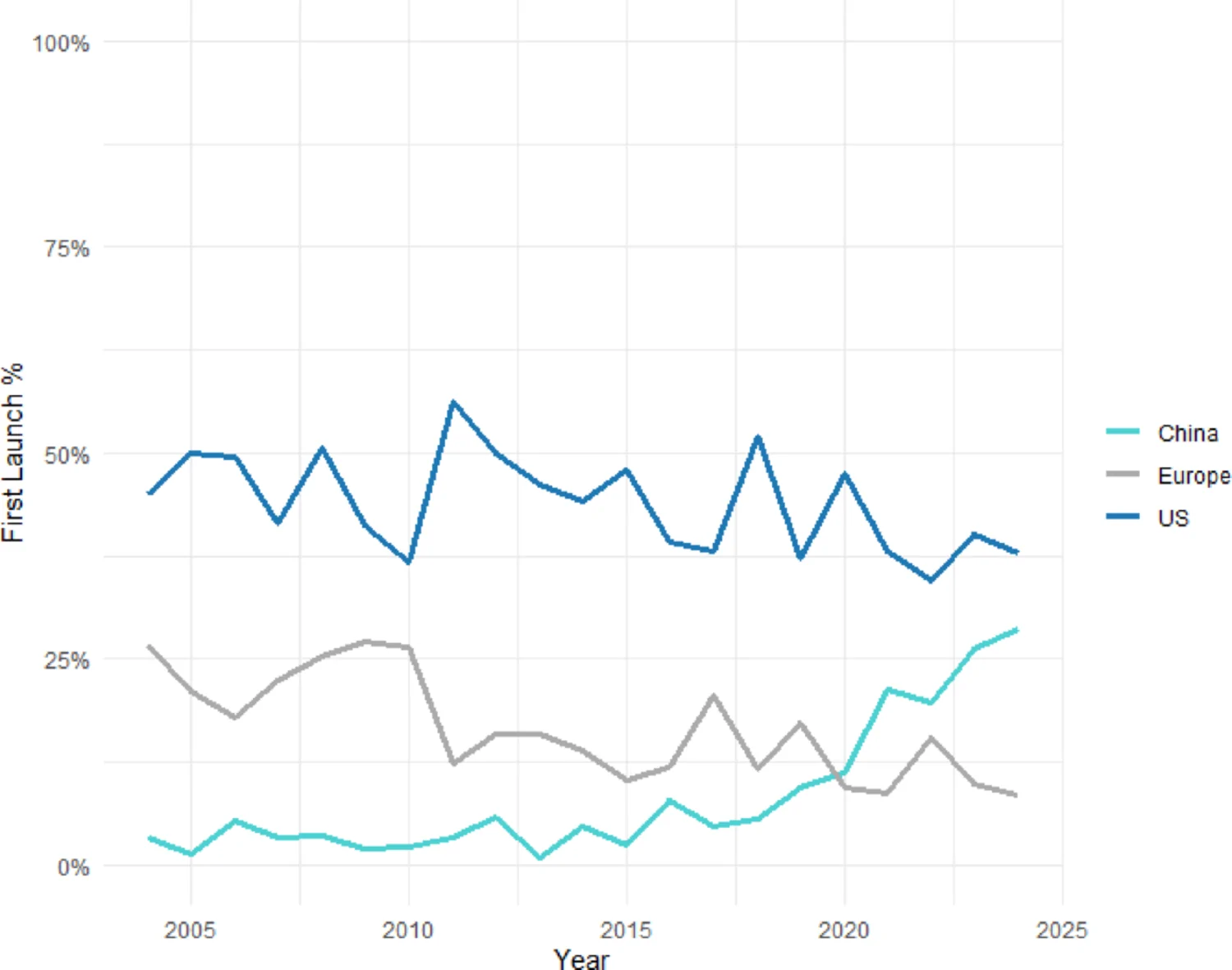
Share of first launches by Region. *Notes* Authors’ analysis of data from Citeline Pharmaprojects database. Totals do not sum to 100% for ease of presentation. Countries accounting for the total remainder included Japan, South Korea, and India. In some years, excluded countries accounted for zero first launches

We identified 2,596 unique first launches in our sample from 2004 through the end of 2024. Of these, 1,120 (43.1%) were first launched in the U.S., 396 (15.3%) were first launched in Europe, and 258 (9.9%) were first launched in China. Countries accounting for the remainder included Japan (9.4%), South Korea (5.5%), and India (4.7%). While both the U.S. and Europe experienced declines in the share of first launches (45.9–37.8% in the U.S.; 26.2–8.5% in Europe), the share of drugs launched first in China grew from 3.3 to 28.7% (Fig. 1).

### First Launches: Disease Category

**Fig. 2:**
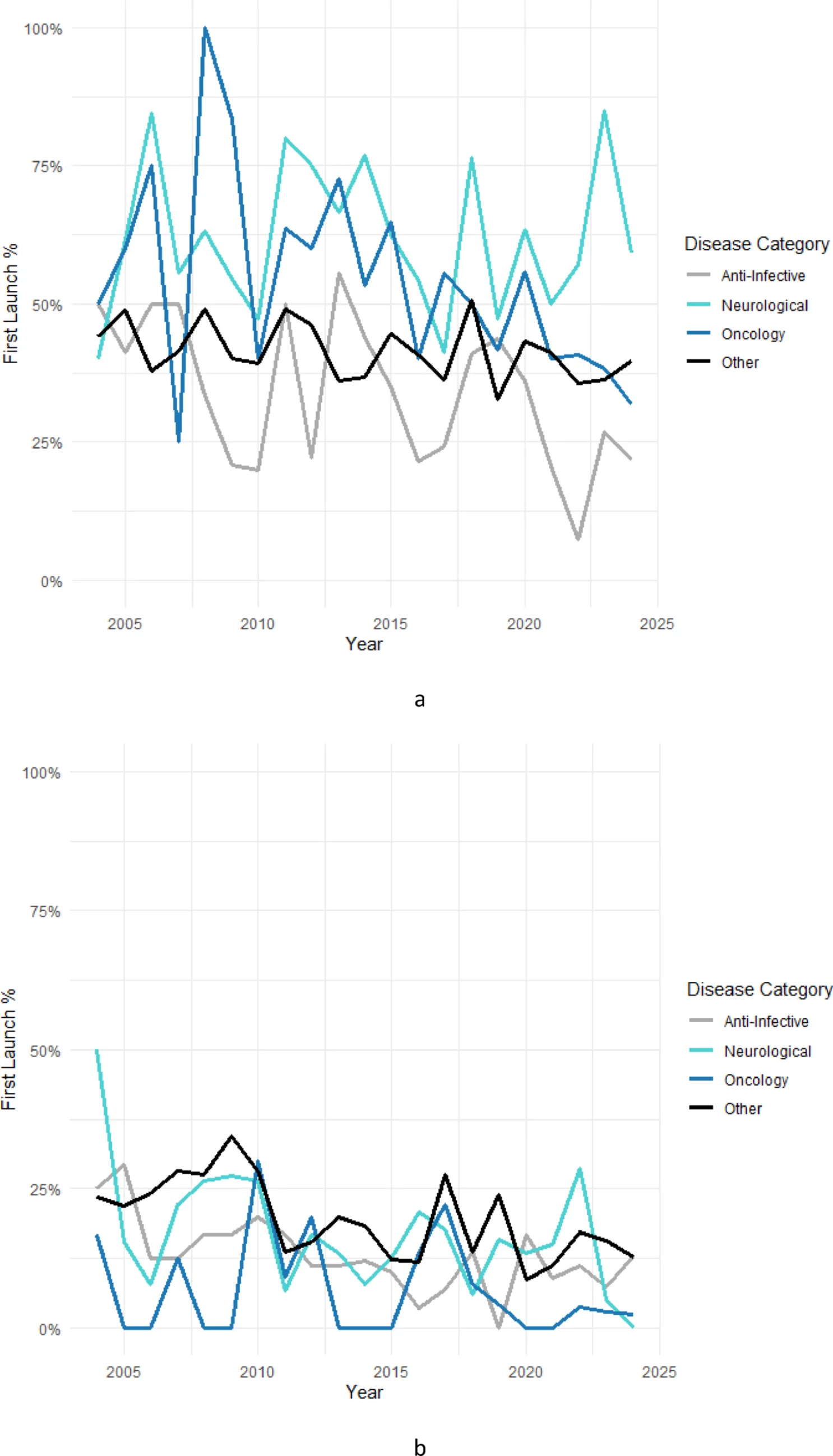

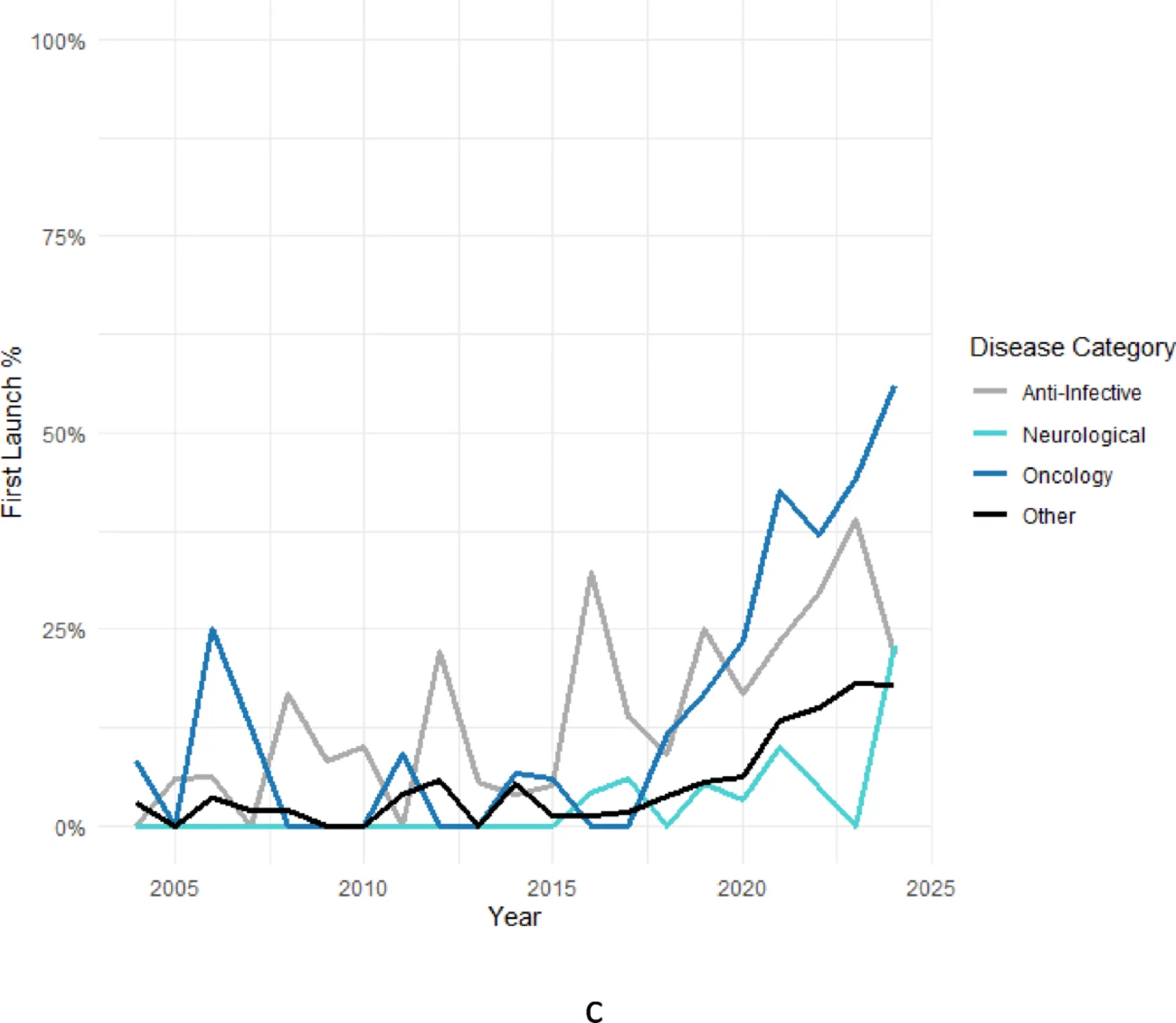
**a** U.S. share of first launches, by disease category. **b** Europe share of first launches, by disease category. **c** China share of first launches, by disease category. *Notes* Authors’ analysis of data from Citeline Pharmaprojects database. Denominator is the total number of drugs launched in a given disease category in a given year. Countries accounting for the total remainder included Japan, South Korea, and India. In some years, excluded countries accounted for zero first launches

Across anti-infective (50.0% in 2004 to 21.7% in 2024) and oncology drugs (50.0% in 2004 to 31.7% in 2024), the U.S. saw a decline in the share of first launches, while neurological drugs and other drugs remained mostly stable (Fig. 2a). Europe saw a decline across all disease categories—25.0–13.0% for anti-infectives, 50–0% for neurological drugs, and 23.5–12.8% for other drugs. Oncology first-launches remained at a low level, often at 0 for some years (Fig. 2b). Across all disease categories, China saw an increase in the share of first launches—from 0 to 21.7% for anti-infectives, 0–22.7% for neurological drugs, 8.3–56.1% for oncology drugs, and 2.9–17.9% for all other drugs (Fig. 2c).

The countries accounting for the remainder did not vary greatly by disease category when compared to the overall findings (e.g., Japan, South Korea, and India) (data not shown).

### First Launches: Biologics and Small Molecules

**Fig. 3:**
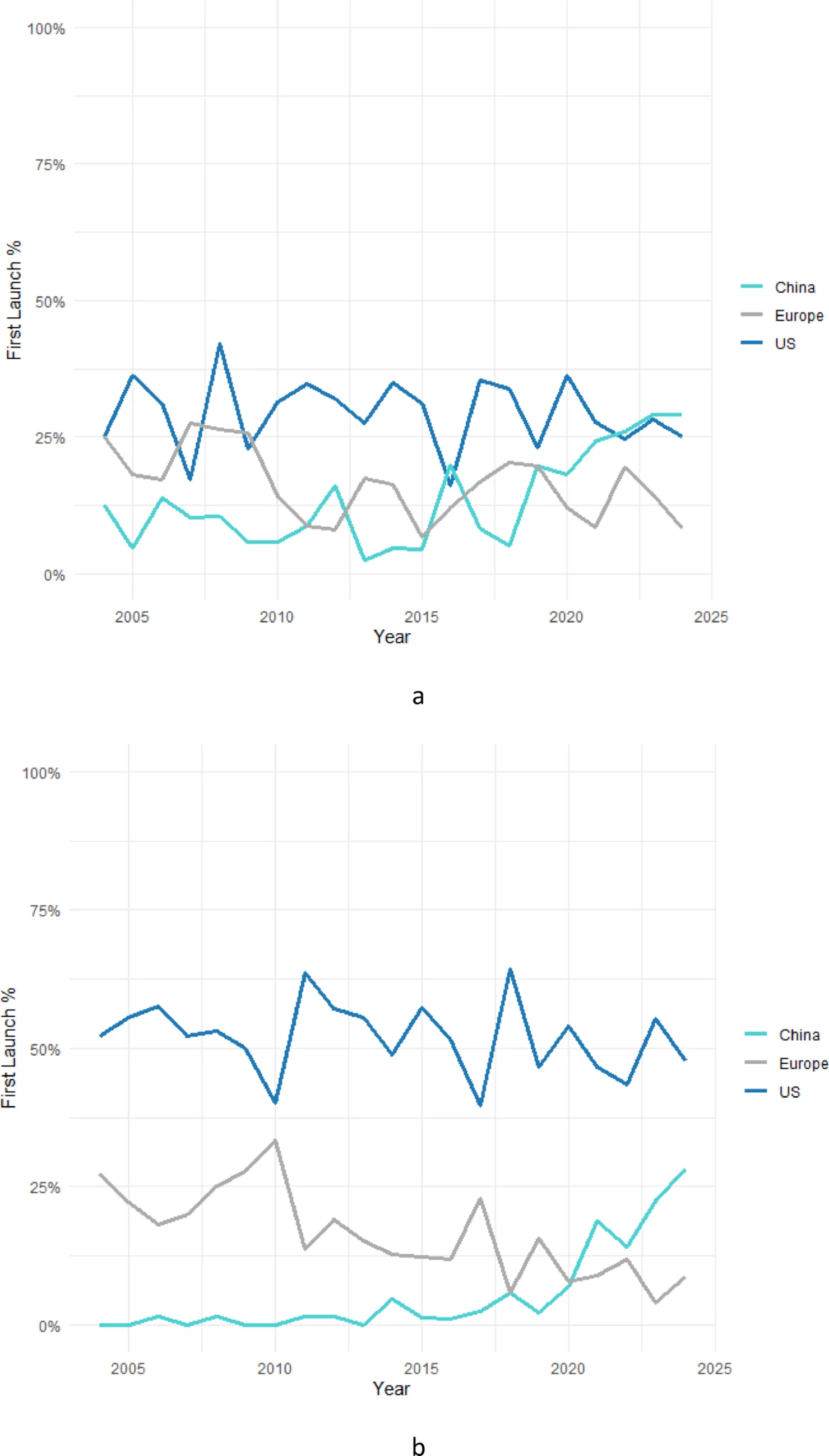
**a** Share of biologic first launches by region. *Notes* Authors’ analysis of data from Citeline Pharmaprojects database. Shares are calculated with all biologics first launched in that year as the denominator. Countries accounting for the total remainder included Japan, South Korea, and India. In some years, excluded countries accounted for zero first launches. **b** Share of small molecule first launches by region. *Notes* Authors’ analysis of data from Citeline Pharmaprojects database. Shares are calculated with all small molecules first launched in that year as the denominator. Countries accounting for the total remainder included Japan, South Korea, and India. In some years, excluded countries accounted for zero first launches

We differentiated overall trends by type of drug: biologic versus small molecule. Among 973 first launches, the U.S. had a larger share of first launches across biologics (28.7%) compared to Europe (15.2%), or China (16.1%). The U.S. share of first launches for biologics remained stable, from 25.0% in 2004 to 25.0% in 2024. Europe’s share of first launches for biologics declined somewhat, from 25.0% in 2004 to 8.3% in 2024. Lastly, China’s share of first launches for biologics increased from 12.5% in 2004 to 29.2% in 2024 (Fig. 3a). The countries accounting for the remainder included India (9.4%), Japan (6.9%), and South Korea (4.9%) (data not shown).

Results were directionally similar for small-molecule drugs. Across 1623 small molecule first launches, the U.S. share of first launches declined slightly from 52.3% in 2004 to 47.8% in 2024. Europe’s share of first launches for small molecule drugs declined from 27.3% in 2004 to 8.7% in 2024, while China’s share of first launches for small molecule drugs increased from 0% in 2004 to 28.3% in 2024 (Fig. 3b). The countries accounting for the remainder included Japan (11.0%), South Korea (5.8%), and India (2.0%) (data not shown).

### Source of First Launches

In addition to the changing landscape of first-launch markets for new drugs, the geographic source of first launches has also changed over the last two decades.

**Fig. 4:**
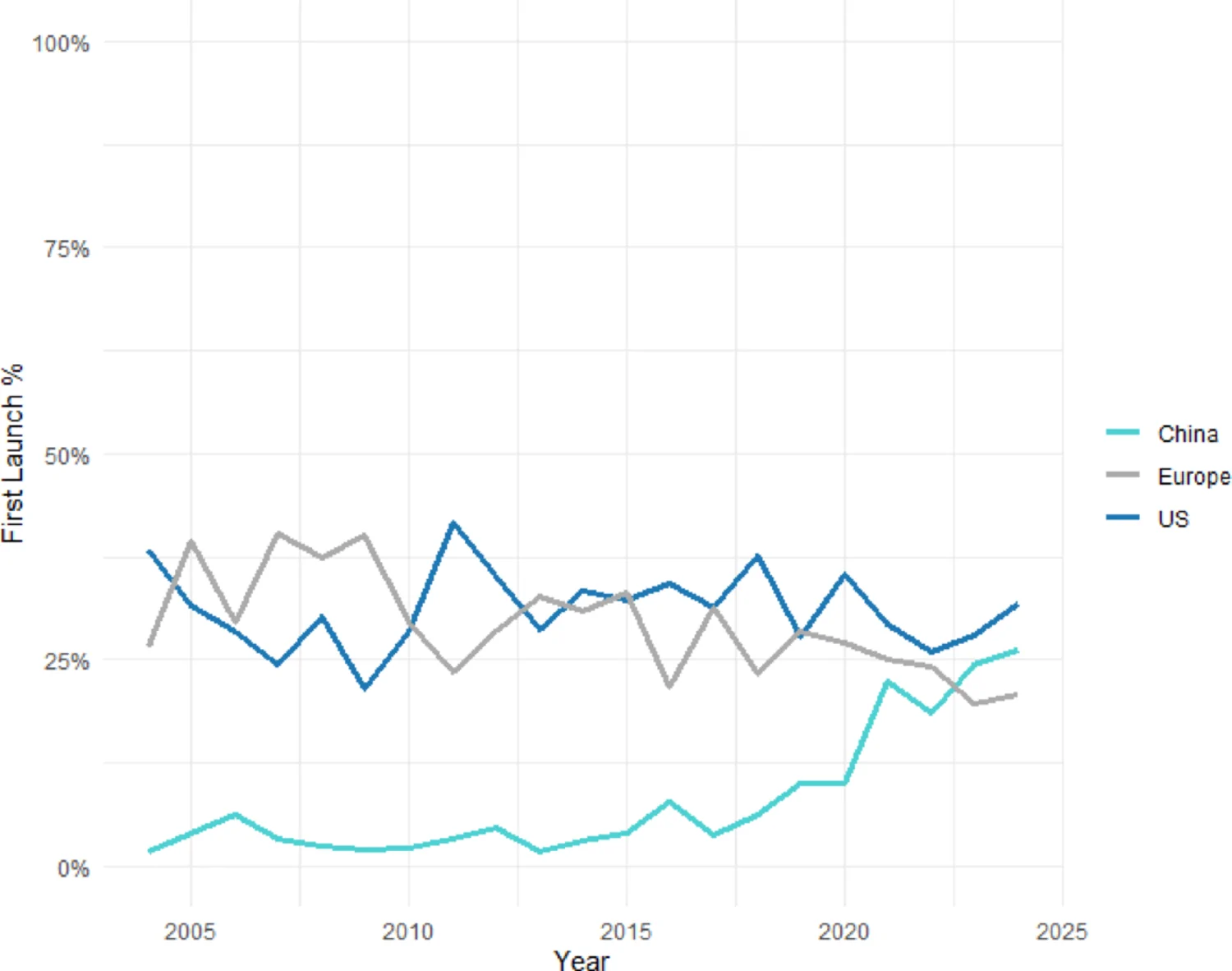
Share of first launches, by company HQ location. *Notes* Authors’ analysis of data from Citeline Pharmaprojects database. Countries represented by the remainder included Japan, South Korea, and India, and generally saw no change or declines in the share of global innovation

Overall, 31.0% of first launches were developed by U.S.-headquartered companies, 28.2% by European companies, and 9.7% by Chinese companies. Countries represented by the remainder included Japan (11.5%), South Korea (6.3%), and India (5.5%), and generally saw no change or declines in the share of global innovation (data not shown). This also declined slightly over time for U.S. (38.3% in 2004, 31.7% in 2024) and European-headquartered companies (26.7% in 2004, 20.7% in 2024), but increased for Chinese companies (1.7% in 2004, 26.2% in 2024) (Fig. 4).

### Source of First Launches: Biologics and Small Molecules

**Fig. 5:**
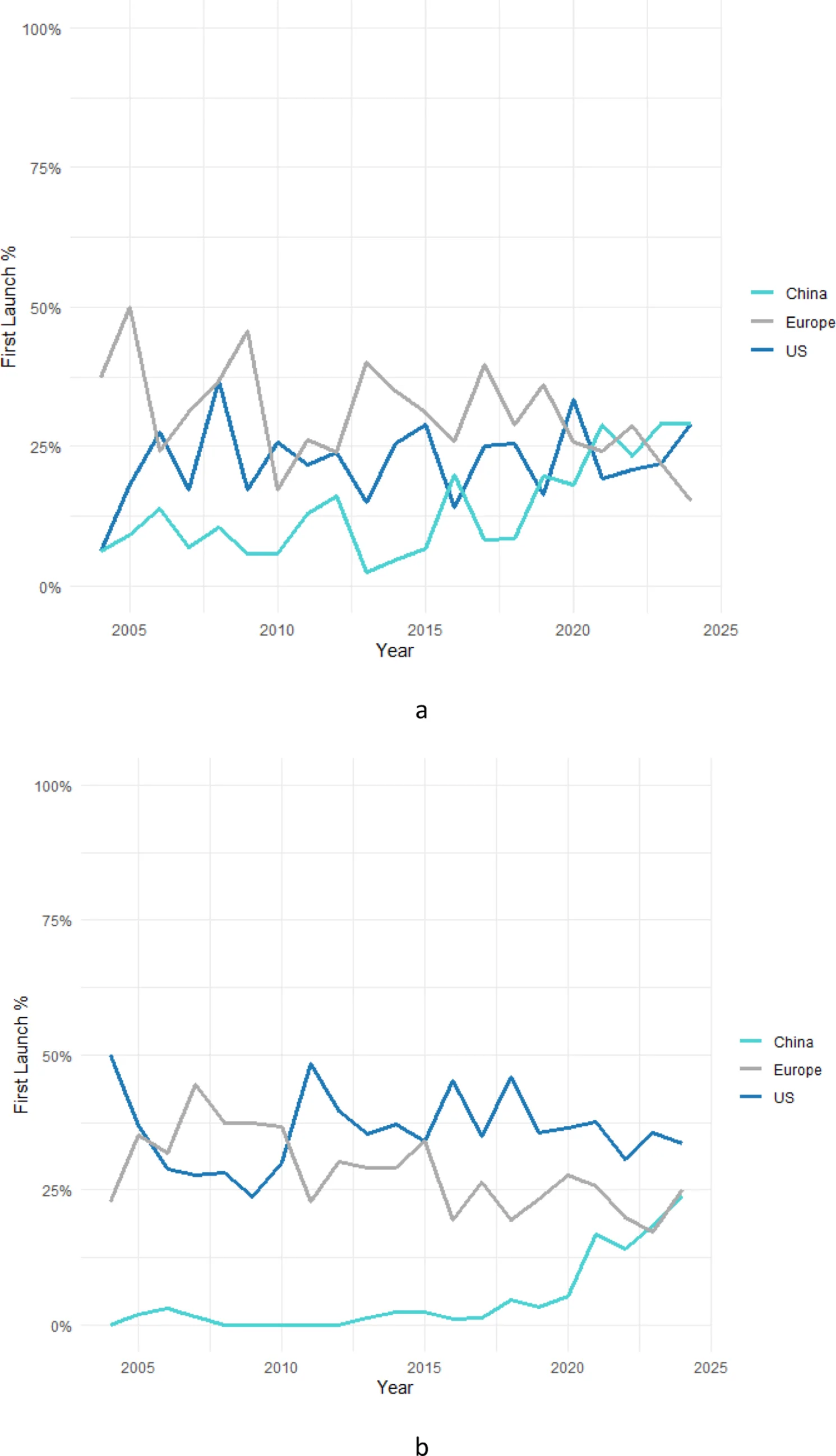
**a** Share of first launches, by company HQ location, biologics. *Notes* Authors’ analysis of data from Citeline Pharmaprojects database. Denominator is the total number of biologic launches in a given year. Countries accounting for the total remainder included Japan, South Korea, and India. In some years, excluded countries accounted for zero first launches. **b** Share of first launches, by company HQ location, small molecules. *Notes* Authors’ analysis of data from Citeline Pharmaprojects database. Denominator is the total number of small molecule launches in a given year. Countries accounting for the total remainder included Japan, South Korea, and India. In some years, excluded countries accounted for zero first launches

From 2004 to 2024, 22.7% of first launch biologics were developed by firms with headquarters (HQs) in the U.S., 28.9% by firms with HQs in Europe, and 16.6% by firms with HQs in China. Trends indicated a stable U.S. share of biologic first launches, a falling European share, and a growing Chinese share (Fig. 5a).

Among small-molecule drugs, 36.0% of first launches were developed by firms with HQs in the U.S., 27.8% by firms with HQs in Europe, and 5.5% by firms with HQs in China. Unlike with biologics, the U.S. share of first launches declined for small molecule drugs as did Europe’s share. The Chinese share of first launches among small molecules increased over this time (Fig. 5b).

## Limitations

This analysis has important limitations. First, biopharmaceutical leadership encompasses many aspects that we could not analyze over the course of 20 years (e.g. jobs, R&D spending, indications, mechanisms of action, etc.). Challenges in the consistent measurement of cross-country measures suggest the need for future efforts to explore these and other measures. Second, within the subset of measures analyzed, the data sources used may have unknown errors (e.g., errors in the classification of variables like disease category and first-launched country). Third, during the study period, there were many policy changes in the U.S. and abroad. Additional research should explore the causes and effects of policy interventions on biopharmaceutical leadership.

**Fig. 6:**
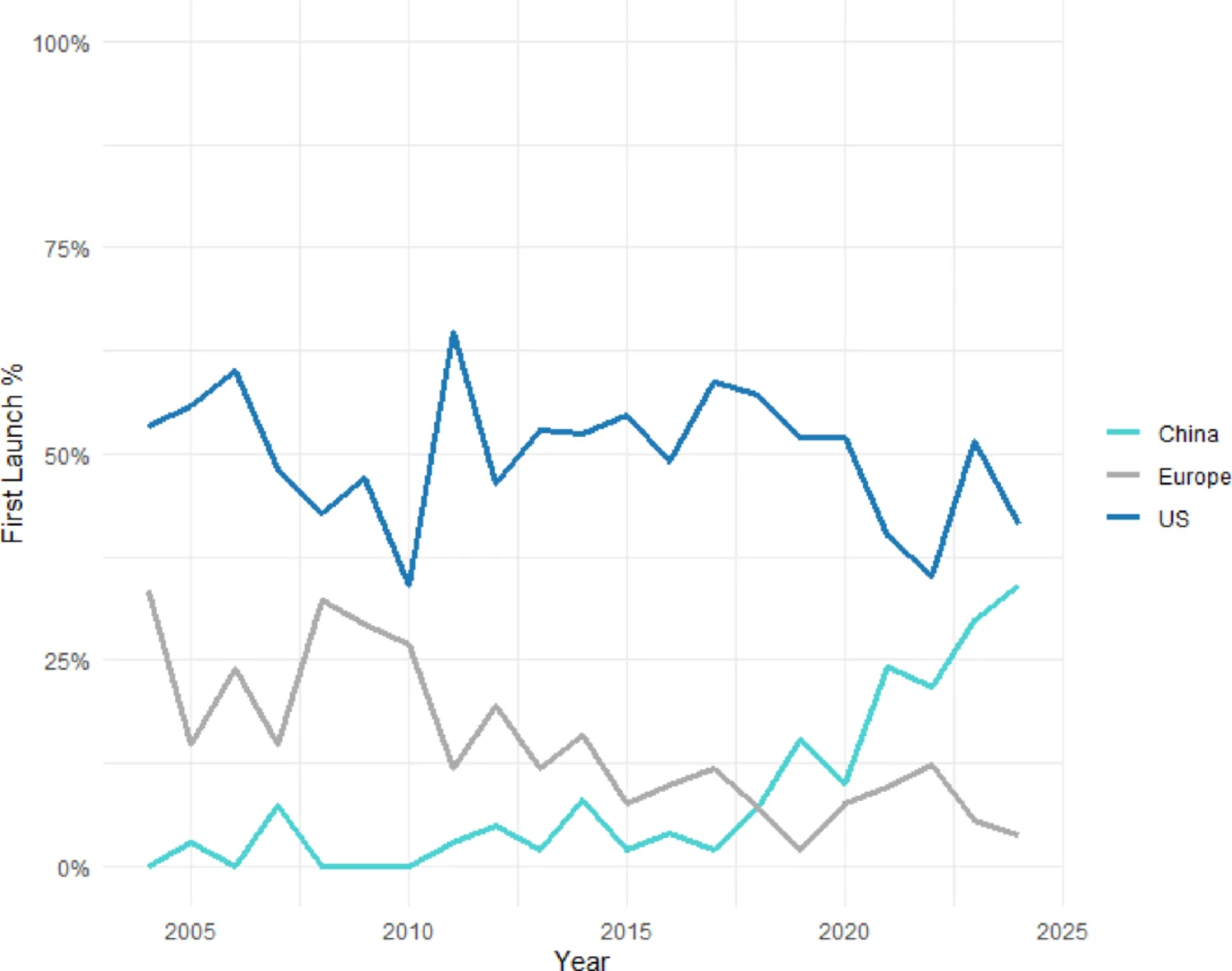
First launch share by region, sensitivity analysis NAS only. *Notes* Authors’ analysis of data from Citeline Pharmaprojects database. Countries accounting for the total remainder included Japan, South Korea, and India. In some years, excluded countries accounted for zero first launches. NAS: New Active Substances

Fourth, because intellectual property tends to be less protected in China, it isn’t clear when drugs developed by Chinese firms or launched first in China represent a derivative of drugs developed or launched elsewhere. Though we attempted to minimize this by focusing on generic drug names, it isn’t possible to fully address this issue. However, to probe this potential concern, we conducted a sensitivity analysis restricting to first launches that are flagged as a “New Active Substance” (NAS) in the Citeline database. Because this flag isn’t complete and won’t capture all true NASs, we chose not to use it in our primary analysis. These sensitivity analyses suggested overall observed trends (Fig. 6) similar to the primary analysis.

## Discussion

Though others have discussed the changing landscape of biopharmaceutical leadership vis-à-vis China [16, 17], this analysis is the first to examine the global change in biopharmaceutical markets over two decades, and describe the characteristics of drugs driving these changes. In this analysis of two decades of biopharmaceutical first launches globally, we identified trends suggesting a decline in the importance of Europe as a first-launch market and increasing relevance of China as both a first-launch market and a source of biopharmaceutical products. This descriptive evidence underscores the view that biopharmaceutical markets and drug development are dynamic and change over time. Though Europe was a substantial source of drug development and an important first-launch market decades ago, this has since shifted.

These findings provide an updated landscape of global biopharmaceutical leadership and are helpful in current debates around U.S. policy, providing a benchmark for future assessments of policy change being deliberated in the U.S. While this analysis supports the view that the U.S remains a leader, the decline in European first-launches suggests that this role is not guaranteed, and supportive policy environments (such as those in China) may be helpful.

Though not examined in this analysis, there are likely many reasons for the growth of first launches and development in China. Some aspects of the health system’s infrastructure likely make clinical trials easier to manage. For instance, the public hospital network in China may make clinical trial patient recruitment easier. Relatedly, the number of hospitals, capital, and staffing has grown, as have the number of pharmaceutical clinical trial institutions. National funding to support research has also grown, making pharmaceutical development less risky. The regulatory environment is also increasingly favorable. Changes such as a reduction in review timelines and expedited programs have likely increased development and first launches.

This evidence presented in this analysis should be considered by policymakers given the rise of first launches occurring in China.

## Data Availability

Data access is restricted through subscription access.
